# Mindfulness-based cognitive therapy added to usual care improves eating behaviors in patients with bulimia nervosa and binge eating disorder by decreasing the cognitive load of words related to body shape, weight, and food

**DOI:** 10.1192/j.eurpsy.2021.2242

**Published:** 2021-10-28

**Authors:** L. Sala, P. Gorwood, C. Vindreau, P. Duriez

**Affiliations:** 1 GHU Paris Psychiatry & Neurosciences, Clinic of Mental Illnesses & Brain Disorders (CMME), 75014 Paris, France; 2 University of Paris, Institute of Psychiatry and Neuroscience of Paris (IPNP), INSERM U1266, 75014 Paris, France

**Keywords:** binge eating disorder, bulimia nervosa, eating disorders, mindfulness-based cognitive therapy

## Abstract

**Background:**

This study aimed to investigate the effectiveness of mindfulness-based cognitive therapy (MBCT) as a complementary approach in patients with bulimia nervosa (BN) or binge eating disorder (BED), and to assess how the reduction of the cognitive load of words related to eating disorders (ED) could constitute an intermediate factor explaining its global efficacy.

**Methods:**

Eighty-eight women and men participated in clinical assessments upon inscription, prior to and following 8-week group MBCT. Mindfulness skills were assessed using the five facet mindfulness questionnaire; eating behaviors were assessed using the Three Factor Eating Questionnaire (TFEQ); comorbid pathologies were assessed using the beck depression index and the state-trait anxiety inventory. The cognitive load of words associated with ED was assessed through a modified version of the Stroop color naming task.

**Results:**

Mindfulness skills improved significantly (*p* < .05) after group MBCT. The improvement of TFEQ scores was accompanied by reduced levels of depressive mood and trait anxiety. The positive impact of MBCT on TFEQ score was directly related to an improvement of the performance in the Stroop task.

**Conclusions:**

MBCT represents an interesting complementary therapy for patients with either BN or BED, at least when cognitive and behavioral domains are concerned. Such efficacy seems to be mediated by the reduction of the cognitive load associated with ED stimuli, which offers a possible explanation of how MBCT could reduce binge-eating behaviors. Other studies are needed, in independent centers, to focus more directly on core symptoms and long-term outcome.

## Introduction

Lifetime prevalence estimates of the adult community are reported as 0.8% for bulimia nervosa (BN) and 1.4% for binge eating disorder (BED) [1]. Individuals with BN or BED lack control over their eating during binge episodes, feeling like they cannot stop eating, or cannot control the quantity of ingested food. Binge eating is defined as eating large amounts of food in a discrete period of time, coupled with a sense of loss of control over one’s eating and emotional distress [2].

Recently, cognitive theories have been proposed to explain binge eating in terms of its antecedents, function, triggers, consequences, and maintaining factors. Despite clinically significant short-term improvements following cognitive behavioral therapy for many individuals with BN and BED, approximately 50% of the patients remain symptomatic in the long term after treatment [3]. It is suggested that a complementary therapy could be useful to treat these patients.

Mindfulness-based interventions are gaining increasing support as efficient approaches to encourage nonjudgmental acceptance of experience [4]. Indeed, mindfulness-based treatment**s** (MBT) emphasize skills and techniques that facilitate increased acceptance of internal experiences (i.e., thoughts, feelings, and physical sensations) [5]. MBT strategies could target the cognitions that initiate and maintain disordered eating [6]. Using mindfulness in the treatment of eating disorders (ED) could help cultivate awareness of internal experiences, facilitate self-acceptance, increase cognitive flexibility, compassion and forgiveness, and generally improve one’s ability to cope adaptatively with emotions [7–10]. Mindfulness-based cognitive therapy (MBCT) could thus have a positive impact on patients with ED as it tackles some of the core aspect**s** of binge eating. Although just a few studies have investigated the application of mindfulness and acceptance-based approaches to disordered eating, early results are promising [5].

Mindfulness is a way of paying attention that is taught through the practice of meditation or other exercises. It is defined as a state of nonjudgmental attention to immediate experience (such as thoughts, emotions, and physical sensations) and an acceptance of moment-to-moment experience [11–14]. Awareness and acceptance of transitory moment allow one to replace automatic thoughts and reactivity to events with conscious and healthier responses [15]. It encourages patients to view emotions and thoughts as transient events that do not require specific behaviors. Mindfulness practices decrease levels of negative affect [16–19]. Their effect is mediated by changes in metacognitions related to emotions and autobiographical memory [20].

Various forms of mindfulness-based interventions have been tested as treatments for individuals with a range of problematic eating behaviors, including emotional or stress-related eating, overeating, and obesity [21]. These interventions typically consist of eight group sessions with a specific topic for each session. MBCT is an extension of Jon Kabat-Zinn’s mindfulness-based stress reduction program.

Studies evaluating the impact of the 8-week mindfulness protocol for patients suffering from ED, and in particular, BN and/or BED, are almost nonexistent. Only Baer et al. [22] have explored an adaptation of MBCT for BED.

Cognitive load can be defined as the mental effort required for an individual to complete a task [23]. The cognitive load theory relies on the assumption that working memory is limited in capacity [24] and that performance drops when the cognitive load increases [25]. Cognitive load can be assessed with the Stroop task [23], which has been used before within the scope of eating behaviors [26, 27]. In this task, subjects are asked to name the color of words [28]. Processing of specifically salient words (such as those related to core aspects of a disorder) imposes a cognitive load that delays color naming [29]. As a result, when words create an attentional bias, naming their color takes longer than when words are neutral [30]. Because food- and body-related stimuli are more salient to individuals with ED [31], the Stroop task with words related to food, body shape, and weight, is an interesting tool to use in this population.

The present study aims to investigate the effectiveness of MBCT, in addition to usual care, in patients with either BN or BED. We hypothesized that MBCT improves eating behaviors as quoted by the Three Factor Eating Questionnaire (TFEQ). Since mindfulness should improve emotion regulation and decrease the negative affect of unpleasant thoughts, we also hypothesized that MBCT decreases the cognitive load of ED-related words. Furthermore, interested in how mindfulness could translate into a higher regulation of eating behaviors, we hypothesized that the efficacy of MBCT on eating behaviors and cognitions was mediated by the reduction of the cognitive load of stressful cues.

## Methods

The research protocol was approved by the Ethics Committees of the hospital and the Ethical Group of the University (UFR SPSE). Each patient received a letter from the Head of the Psychiatry Department confirming the researchers involved, the objectives of the study, the clinical protocol, and data anonymity prior to the signature of an informed consent form that systematically confirmed their participation. Subjects received no form of payment for participating in the research.

### Participants

Eighty-eight patients attending a day hospital at a university hospital specialized in the treatment of ED were enrolled over a 3¾-year period between October 2014 and June 2018. Mean age on enrolment was 30.8 years (standard deviation [SD] = 8.5, range: 19–68), 88% (*n* = 44) were female, and 75% (*n* = 66, 1 male) were diagnosed as suffering from BN. Mean body mass index (BMI) was 23.5 (SD = 5.5, range: 16–40).

Patients were evaluated in a clinical interview with a senior psychiatrist to classify their ED based on The Diagnostic and Statistical Manual of Mental Disorders, fifth edition criteria [2]. Subjects diagnosed with either BN or BED were included in the study. Exclusion criteria included patients diagnosed with anorexia nervosa (AN), schizophrenia, bipolar disorders, or addiction. Five patients with BN had a BMI below 18.5 (but above 16.5). Nutritional interventions and treatments were allowed throughout the experiment.

Patients were assigned to one of six groups, containing from 9 to 12 participants. Some of them were initially placed on a waiting list (WL) prior to participating in the MBCT protocol, the waiting time for which varied from 2 to 6 months. The WL phase was not systematic in order to ensure adequate and coherent group sizes: 54% (*n* = 33) of patients undertook the WL phase, during which they received usual care. Seventeen patients failed to complete tests and questionnaires for more than one evaluation timepoint; consequently, the final sample size was 71 patients. It was decided to include male patients from the outset, even though the proportion of the overall sample they could represent was unknown.

### MBCT protocol

During the MBCT, in addition to usual care, patients took part in eight 2-h weekly sessions over a 2-month duration. Sessions were conducted by a senior instructor and psychologist with more than 14 years of MBCT practice. The program closely followed the standard program conceived by Segal et al. [32], but the psychoeducation was tailored to suit subjects presenting ED rather than depression. Similarly, different cognitive tools were used and the duration of the meditation practices was reduced from 45 to 30 min. The first phase (sessions 1–4) focused on paying attention to the present moment by learning to observe one’s mental dispersion; the second phase (sessions 5–8) focused on a recent problem from daily life in order to develop a different relationship with the unfavorable event and the associated emotions.

### Clinical assessments

Tests and questionnaires were completed by patients at three timepoints: upon inscription on WL (T_0_), prior to group MBCT (T_1_), and at the end of the protocol (T_2_). Patients’ weight and height were measured to determine their BMI (weight/height²).

The evolution of mindfulness skills was measured using the Five-Facets Mindfulness Questionnaire (FFMQ), a 39-item self-completed questionnaire measuring the five facets of mindfulness: Observing (8 items), Describing (8 items), Acting with awareness (8 items), Nonjudgmental (8 items), and Nonreactive (7 items). Items are rated on 5-point Likert scales (1 = never or very rarely true to 5 = very often or always true), each facet score ranges from 8 to 40, except for the nonreactive facet which ranges from 7 to 35. For each facet, higher scores indicate higher levels of mindfulness [33].

Eating behaviors were evaluated using the TFEQ, a 51-item self-assessment scale assessing three factors: Cognitive restraint (CR) (21 items), Disinhibition (16 items), and Hunger (14 items). Each item is scored either 0 or 1. Minimum scores for the three factors are 0, maximum scores 21, 16, and 14, respectively. Higher scores indicate higher levels of restrained eating, disinhibited eating, and predisposition to hunger [34]. The TFEQ has psychometric support including predictive validity [35].

The Beck Depression Inventory (BDI) [36] and the State-Trait Anxiety Inventory (STAI) [37] were administered to assess comorbid depressive and anxiety symptoms respectively. The BDI is a 21-question multiple-choice self-report inventory. Each question has four possible responses, ranging in intensity, scored from 0 to 3. Higher scores indicate more severe depressive symptoms. The STAI (form Y) is a 40-item self-evaluation questionnaire for assessing trait anxiety (20 items) and state anxiety (20 items). All items are rated on 4-point scales (e.g., from “Almost Never” to “Almost Always”). Higher scores indicate greater anxiety.

### Eating disorder Stroop task

Following a search of the international literature, we chose a modified version of the Stroop task published by Cooper and Todd [27], who validated the adaptation of the test to ED. A French translation of this ED-specific Stroop task was developed following consultation with the corresponding author.

Ten cards were generated. Each card was made up of 25 words printed on a white background in 5 rows of 5 words. Each word was printed in one of five colors: red, green, black, blue, or yellow. Each of the five stimulus words was repeated five times. In each block of five words, each of the five words and each of the five colors occurred once.

The 10 cards were organized into 5 pairs and presented in a fully balanced design. In each pair, the target card followed the control card. The pairings were: congruent words and colors versus incongruent words and colors; transport versus food; household objects versus weight; nature versus body shape; and communication versus depression.

Patients were instructed to name the color of the ink in which each word was written as quickly as possible and to correct any errors immediately after their occurrence. The time taken to name the color of all the words on each card was recorded using a digital stopwatch. Subsequently, the total time taken to name all neutral words (six cards: congruent colors, incongruent colors, transport, objects, nature, and communication) and all ED-related words (three cards: food, weight, and shape) were calculated.

The test was administered at timepoints T_0_, T_1_, and T_2_, following the clinical interview with the senior instructor and prior to completion of the clinical assessments.

### Statistical analysis

All analyses, except path analyses, were performed with the PASW Statistics18 software. Normal distributions of variables were checked using Kolmogorov–Smirnov test prior to analysis. Score changes were calculated for the WL period (difference between timepoints T_1_ and T_0_) and for the duration of MBCT (difference between timepoints T_2_ and T_1_). *T*-tests were performed for statistical comparison between WL and MBCT. Homogeneity of variances was confirmed using the Levene statistic. The significance level was set at *p ≤* 0.05.

Multivariate approaches were performed by logistic regression to evaluate the role of any potentially contaminating factors. This analysis used the condition MBCT versus WL as the dependent variable, and the following independent variables: change of TFEQ scores as the expected improvement, and changes of BDI (depression) and STAI-State (anxiety) scores as potential confounders. The hypothesis was that any improvement of the TFEQ score as a parameter was independent of any improvement following MBCT, and not merely a reflection of an improvement of mood or anxiety scores.

Path analyses were conducted using PROCESS statistics for SPSS v3.5, to assess the direct impact of MBCT on the improvement of the TFEQ score and the mediating effect of Stroop performance.

## Results

After MBCT, four out of the five facets of the FFMQ showed statistically significant improvements: Observing (*t* = 2.34, *p* = 0.01), Describing (*t* =1.71, *p* = 0.05), Nonjudging of inner experience (*t* = 1.63, *p* = 0.05), and Nonreactivity to inner experience (*t* = 1.77, *p* = 0.04). The only facet not to improve significantly was Acting with awareness (*t* = 1.39, *p* = 0.08). The effect sizes were small to moderate (Table 1).

**Table 1.** Comparison of mindfulness capacities (FFMQ), eating behavior (TFEQ), depressive symptoms (BDI), trait and state anxiety (STAI), and emotional reactivity (modified Stroop test for ED) in 61 patients treated for an ED following inscription on a waiting list (WL) and before and after 8 weeks of group MBCT.

Parameters		Baseline values at study enrolment		WL		MBCT		Difference from 0 of			
		(T_0_)		(T_1_–T_0_) (*N* = 33)		(T_2_–T_1_) (*N* = 52)		(T_2_–T_1_)–(T_1_–T_0_)			
		Mean	SD	Mean	SD	Mean	SD	*t*	df	*p-value*	Cohen’s *d*
FFMQ	Observing	22.2	6.58	−0.58	3.40	1.71	4.88	2.34	82	0.01*	0.53
	Describing	21.4	6.86	−0.03	3.38	1.59	4.71	1.71	82	0.05*	0.39
	Awareness^a^	22.1	6.49	−0.76	5.68	1.04	5.86	1.39	82	0.08	0.31
	Nonjudging^b^	21.3	5.88	0.00	5.23	2.27	6.79	1.63	82	0.05	0.37
	Nonreactivity^c^	12.4	3.14	0.51	2.25	1.86	4.67	1.77	82	0.04*	0.35
TFEQ	Cognitive restraint	12.1	4.66	−0.19	2.28	−1.30	2.76	1.90	82	0.06	−0.43
	Disinhibition	11.1	3.71	0.09	1.51	−1.00	2.76	2.31	82	0.02*	−0.46
	Hunger	7.2	4.27	0.56	2.44	−0.64	2.44	2.18	82	0.03*	−0.50
	Total	30.3	7.02	0.47	4.70	−2.92	6.02	2.72	82	0.01^**^	−0.61
BDI		11.3	4.81	−0.03	4.93	−2.50	4.92	2.55	82	0.01^**^	−0.51
STAI-State		63.2	10.90	−0.51	11.90	−3.69	12.40	1.18	82	0.18	−0.26
STAI-Trait		64.7	9.07	−0.39	7.11	−4.52	9.02	2.35	82	0.01*	−0.49
Stroop words	Congruent colors	15.4	3.84	−2.48	3.71	−1.17	3.62	1.60	82	0.06	0.36
	Incongruent colors	25.2	10.20	−2.58	7.54	−3.66	5.08	0.72	82	0.24	−0.18
	Transport	18.0	5.23	−1.48	2.36	−0.74	1.98	1.50	82	0.07	0.35
	Objects	16.7	3.88	0.17	2.09	−1.22	2.85	2.59	82	0.01^**^	−0.53
	Nature	18.1	4.19	−0.80	2.37	−0.93	2.61	0.24	82	0.41	−0.05
	Communication	16.1	5.52	0.41	2.39	−0.81	2.80	2.14	82	0.02*	−0.46
	All neutral words	84.3	19.00	−4.18	6.91	−4.87	7.41	0.44	82	0.33	−0.10
	Food	17.8	3.13	−0.19	3.25	−1.50	3.04	1.86	82	0.03*	−0.43
	Weight	20	6.09	−1.01	3.62	−1.78	2.66	1.05	82	0.15	−0.26
	Shape	18.5	4.48	−0.87	2.25	−1.83	2.40	1.87	82	0.03*	−0.41
	All ED words	56.2	13.00	−2.06	5.83	−5.11	5.84	2.35	82	0.01*	−0.53
	Depression-related words	18.5	4.95	−0.95	2.50	−0.94	3.57	0.02	82	0.49	<0.01

Abbreviations: BMI, body mass index; BDI, Beck depression inventory; FFMQ, five facets mindfulness questionnaire; MBCT, mindfulness-based cognitive therapy; STAI, state-trait anxiety inventory; TFEQ, three-factor eating questionnaire; WL, waiting list.

a Acting with awareness.

b Nonjudging of inner experience.

c Nonreactivity to inner experience.

* *p* ≤ 0.05.

** *p* ≤ 0.01.

We observed a significant improvement in TFEQ results during MBCT, with a moderate total effect size (Table 1). The level of depressive mood and the trait condition of anxiety also significantly improved after MBCT and not so following inscription on the WL (Table 1). Furthermore, when assessing the efficacy of MBCT on TFEQ score, we observed no impact of the sessions (*F* = 0.259, *p* = 0.933), gender (*t* = 1.196, *p* = 0.119), diagnoses (*F* = 0.231, *p* = 0.633), age (*r* = 0.01, *p* = 0.969), or baseline BMI (*r* = −0.075, *p* = 0.603).

The logistic regression analysis showed that only TFEQ improvement was significant (Wald *χ*² = 4.65, df = 1, *p* = 0.03) following MBCT, while BDI (Wald *χ*² = 2.93, df = 1, *p* = 0.09) and state anxiety (Wald *χ*² = 0.253, df = 1, *p* = 0.62) were not.

In the Stroop task, reaction times for ED-related words were shortened (*t* = 2.24, *p* = 0.01, *d* = 0.52) by MBCT, whereas those for neutral (*t* = 0.349, *p* = 0.36, *d* = 0.08) and mood (*t* = 0.015, *p* = 0.49, *d* < 0.01) words were not (Table 1).

**Figure 1.**
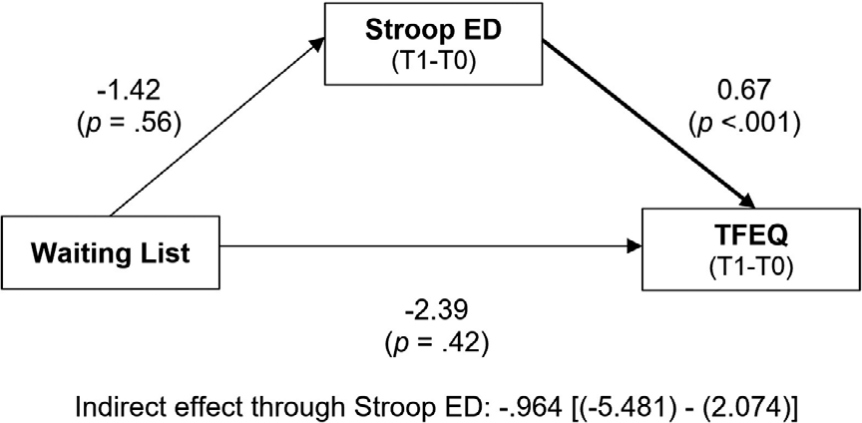
Path analysis of the impact of the “waiting list” on “TFEQ” score, directly, and through its impact on the “Stroop ED” test (for words related to eating disorders). Changes for both TFEQ score and performance in the emotional Stroop task associated with the waiting list correspond to the difference between timepoints T0 and T1. Bold arrows indicate significant paths (p<.05). There was no direct effect of the waiting list neither on the TFEQ score nor on emotional Stroop performance for ED-related words. ED symptoms improvement was significantly predicted by emotional Stroop performance.

The path analysis on the effect of being tested twice, before and after the WL (control intervention, Figure 1), showed no direct effect neither on TFEQ score (*t* = −0.804, *p* = 0.42) nor on reaction times in the Stroop task for ED-related words (*t* = −0.600, *p* = 0.56). TFEQ score was significantly predicted by reaction times (*t* = 5.545, *p* < 0.001). The bootstrapping indirect effect of WL on TFEQ score improvement through Stroop performance was not significant (CI 95% [(−5.481) − (+2.074)]).

**Figure 2.**
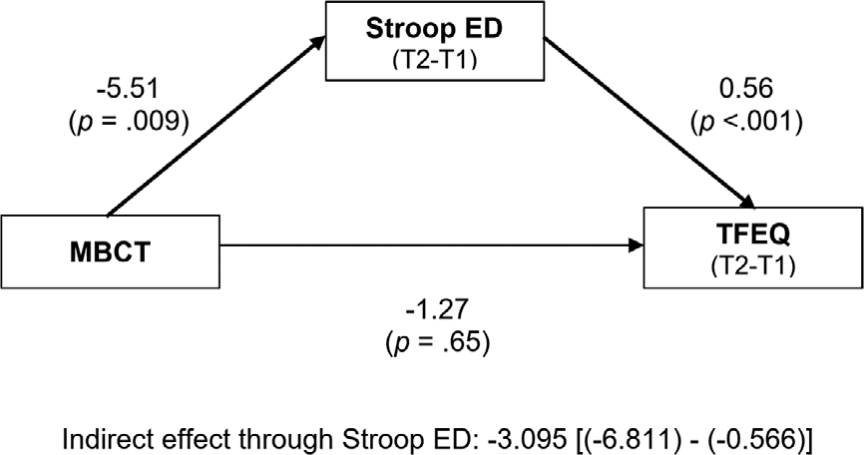
Path analysis of the impact of the mindfulness-based cognitive therapy (“MBCT”) on “TFEQ” score, directly, and through its impact on the “Stroop ED” test (for words related to eating disorders). Changes for both TFEQ score and performance in the emotional Stroop task associated with MBCT correspond to the difference between timepoints T1 and T2. Bold arrows indicate significant paths (p<.05). There was no direct effect of MBCT on the TFEQ score but a strong effect on emotional Stroop performance for ED-related words. ED symptoms improvement was significantly predicted by emotional Stroop performance.

A similar path analysis performed before and after MBCT (studied intervention, Figure 2) showed no residual direct effect of MBCT on TFEQ score (*t* = −0.449, *p* = 0.65), but a strong effect on reaction times in the Stroop task for ED-related words (*t* = 4.231, *p* = 0.009). TFEQ score improvement was significantly mediated by the improvement of reaction times (*F* = 10.227, *p* < 0.001). The bootstrapping indirect effect of MBCT on TFEQ score improvement through Stroop performance was significant (CI 95% [(−6.811) − (−0.566)]).

None of the preceding results were changed when the five patients with a BMI below 18.5 were excluded (data not shown).

## Discussion

The impact of MBCT was assessed on patients with either BN or BED. We found that MBCT significantly improved mindfulness, TFEQ scores and reaction times in the Stroop task. It also had a significant positive impact on depression and trait anxiety. The improvement of the three factors of ED tested by the TFEQ (dietary restriction, disinhibition, and hunger) was mediated by the reduction of reaction times in the Stroop task for words related to food, weight, and shape. These results support the idea that MBCT could induce positive changes in disordered eating, such as improving eating behaviors and, in parallel and independently, the anxiety and depressive symptoms tested herein.

Our study confirms the few results published in literature regarding the impact of MBCT for patients with BN or BED. Previous reviews have suggested that mindfulness-based interventions are effective in reducing symptoms of ED [7, 38, 39] and could reduce binge episodes and dichotomous thinking, body image concern, and emotional eating [7, 21]. Similarly, our results concerning improvement in mindfulness, anxiety, and external-based eating are consistent with those of Daubenmier et al. [40]. However, the sample tested herein is larger than the subject populations comprised in most of these studies.

In the original version of the Stroop task [28], subjects are asked to name the color of color names written in congruent or incongruent colors. This version of the task measures the ability to refrain from reading the color name and to name the color of the ink. In the modified version that we used [27], the difficulty does not arise from congruent or incongruent colors, but depends on the salience of the words [30]. Indeed, words related to eating disorder have an increased cognitive load, which interferes with the ability to complete the task [29]. Cooper and Todd, whose version of the Stroop test we used, reported that patients with ED took longer to color name words related to their concerns with eating, weight, and body shape than neutral words [27]. In the present study, we found that patients were faster to color name ED-related words post-MBCT than pre-MBCT. This suggests that MBCT efficiently decreased their concerns/biases with food, weight, and body shape and, by doing so, reduced the salience and the resulting cognitive load of ED-related words.

Impulsivity promotes binge-eating behaviors in both BN [41] and BED [42]. Mindfulness and impulsivity are generally negatively correlated; indeed, Urgency (one of the facets of impulsivity) was reported to be negatively and strongly associated with Acting with Awareness, Nonreactivity, and Nonjudgment [43]. MBCT could therefore reduce binge-eating behaviors by improving mindfulness and consequently decrease impulsiveness. An alternative explanation could rather propose that MBCT targets executive control and/or emotion regulation. In a study devoted to bereavement and depression, MBCT facilitated the executive control function by alleviating the emotional interferences over the cognitive functions, suggesting that the MBCT intervention significantly improved both executive control and emotion regulation [44]. In the present study, however, the co-occurring improvement of mood was not involved in the improvement of eating behaviors, while we observed a significant role of the reduction of the cognitive load of ED-related words. This suggests that cognitions (inhibitory control) rather than emotions are the leverage of MBCT efficacy in ED.

As mindfulness-based interventions primarily aim to facilitate self-acceptance, improve the ability to cope with emotions and decrease levels of negative affect [7–10, 16–19], it is not surprising that MBCT was associated with a reduction of anxiety and depressive symptoms. This constitutes another significant advantage of using MBCT as a complementary therapy in patients with ED who often suffer from depression and anxiety [45, 46].

Our findings are in accordance with the strength model of self-regulation [47–49]. This model posits that individuals have a limited capacity to regulate certain states (e.g., affect and hunger), and can benefit from mindfulness interventions to continue to increase their capacity for self-regulation, affective stability and flexibility, coping skills, and reduced reactivity toward stress-induced bulimic compulsions.

Four limitations should be considered in the present study.

First, the fact that just 54% of patients completed the WL phase prior to MBCT reduced the statistical power to detect an effect in this specific group and in the global sample. The inclusion of patients in the WL was stopped at least 4 weeks before the first MBCT group started, in order to ensure a sufficiently long waiting time, which is at least 1 month. This strategy was decided to reduce the negative consequences of the protocol in the treatment proposed in our center, and reduce the risk of biases associated with the inclusion of just volunteers. Our WL multiple baseline therefore cannot be considered as random, but probably facilitated the low attrition rate (22%) that we obtained.

Second, WL conditions may act as a nocebo in psychotherapy trials [50], and this might be amplified by our multiple baselines. The suspicion that this might lead to different effect size estimates did not form part of this study. Future research should address this weakness, notably by preplanning sensitivity analyses and performing post hoc analyses on findings of significant differences in order to adjust for potential publication bias.

Third, our tests lacked an evaluation of general psychopathology. Regarding the neuropsychological assessment, we only included in our study the Stroop test, but additional measures regarding memory and executive functions would have been useful. The Stroop task has some well-known limitations, such as variable scoring methods [51], a contaminating role of optometric [52], presence of dyslexia [53] or depressive disorder [54], honesty of responses [55] or even gum-chewing [56]. Furthermore, the Stroop task probably tested variable dimensions of cognitive control [57]. Knowing that pupil size increases as task demand rises, the observation of a steep increase of pupil size when reading incongruent distractors is reassuring [58]. Likewise, finding in our sample that mood and anxiety improved independently from reaction times is also reassuring. Associating different neurocognitive tests with the Stroop task would help define the neurocognitive aspects which could explain the positive effect of MBCT in ED.

Fourth, path analyses attribute the impact of an intervention on one intermediate factor to explain a global effect on another. While detecting that Stroop performance mediates the improvement of the TFEQ score is interesting, these analyses do not take into account that such effect probably differs from one patient to another. In other words, MBCT probably has more obvious efficacy in specific patient subgroups, and our sample was too small to be able to define them. In the same line, the instruments used in the present protocol only reflect some aspects of eating, mood and anxiety disorders, and cannot be considered representative of the large heterogeneity of patients having these disorders.

In conclusion, while the current study presents interesting findings on the role of group MBCT and its relationship with eating behaviors, future research will be important to include biological parameters and other tests of cognitive flexibility and functioning.

## Data Availability

The data that support the findings of this study are not available.
